# Supplementary material for: A protocol for single nucleus RNA-seq from frozen skeletal muscle
Source: Life Sci Alliance. 2023 Mar 13;6(5):e202201806. doi: 10.26508/lsa.202201806 (PMC10011611; doi:10.26508/lsa.202201806)
Supplement: Supplementary file 1 [file LSA-2022-01806_TableS1.docx]

**Table S1**

| avg_log2FC | pct.1 | pct.2 | p_val_adj | cluster | gene |
| --- | --- | --- | --- | --- | --- |
| 3.37309107 | 0.993 | 0.329 | 0 | Muscle Type I | ATP2A2 |
| 3.02079453 | 0.967 | 0.196 | 0 | Muscle Type I | MYH7 |
| 2.93937582 | 0.987 | 0.261 | 0 | Muscle Type I | TPM3 |
| 2.93320351 | 0.995 | 0.315 | 0 | Muscle Type I | TNNT1 |
| 2.57924759 | 0.843 | 0.13 | 0 | Muscle Type I | LGR5 |
| 2.29337341 | 0.823 | 0.076 | 0 | Muscle Type I | MYH7B |
| 2.26546446 | 0.949 | 0.386 | 0 | Muscle Type I | XPO4 |
| 1.99867785 | 0.681 | 0.058 | 0 | Muscle Type I | TECRL |
| 1.93336118 | 0.886 | 0.205 | 0 | Muscle Type I | TNNI1 |
| 1.74855472 | 0.84 | 0.256 | 0 | Muscle Type I | MYL2 |
| 1.74586286 | 0.802 | 0.213 | 0 | Muscle Type I | ESRRG |
| 1.73662404 | 0.847 | 0.205 | 0 | Muscle Type I | MYOM3 |
| 1.64348375 | 0.664 | 0.131 | 0 | Muscle Type I | ZNF385B |
| 1.60048147 | 0.49 | 0.062 | 0 | Muscle Type I | MYOZ2 |
| 1.40813378 | 0.914 | 0.611 | 0 | Muscle Type I | RIF1 |
| 1.38405268 | 0.906 | 0.396 | 0 | Muscle Type I | CD36 |
| 1.36142995 | 0.913 | 0.467 | 0 | Muscle Type I | USP54 |
| 1.27897541 | 0.867 | 0.421 | 0 | Muscle Type I | USP13 |
| 1.26564657 | 0.621 | 0.149 | 0 | Muscle Type I | ALPK2 |
| 1.26357013 | 0.411 | 0.073 | 0 | Muscle Type I | CCR3 |
| 1.25020171 | 0.504 | 0.056 | 0 | Muscle Type I | MYLK3 |
| 1.22704469 | 0.999 | 0.82 | 0 | Muscle Type I | NEB |
| 1.22515039 | 0.585 | 0.113 | 0 | Muscle Type I | SLC1A3 |
| 1.21849655 | 0.87 | 0.424 | 0 | Muscle Type I | RCAN2 |
| 1.21722634 | 0.459 | 0.134 | 0 | Muscle Type I | CA3 |
| 1.21569324 | 0.612 | 0.24 | 0 | Muscle Type I | GAS2 |
| 1.19240221 | 0.87 | 0.398 | 0 | Muscle Type I | TPD52L1 |
| 1.17701808 | 0.994 | 0.642 | 0 | Muscle Type I | MYOM1 |
| 1.17447382 | 0.298 | 0.028 | 0 | Muscle Type I | AC099066.2 |
| 1.17366624 | 0.516 | 0.123 | 0 | Muscle Type I | ASB18 |
| 1.14626553 | 0.749 | 0.384 | 0 | Muscle Type I | CRIM1 |
| 1.14374339 | 0.761 | 0.307 | 0 | Muscle Type I | LMOD2 |
| 1.14179642 | 0.841 | 0.448 | 0 | Muscle Type I | EXOC6 |
| 1.13445479 | 0.819 | 0.38 | 0 | Muscle Type I | AC106791.1 |
| 1.12348106 | 0.833 | 0.372 | 0 | Muscle Type I | KCNMA1 |
| 1.10462983 | 0.824 | 0.345 | 0 | Muscle Type I | LRRC39 |
| 1.1041716 | 0.582 | 0.21 | 0 | Muscle Type I | NEK10 |
| 1.09926639 | 0.555 | 0.125 | 0 | Muscle Type I | ATP1B4 |
| 1.09738811 | 0.904 | 0.436 | 0 | Muscle Type I | LRRC2 |
| 1.09291407 | 0.953 | 0.668 | 0 | Muscle Type I | TTN-AS1 |
| 1.08000308 | 0.988 | 0.602 | 0 | Muscle Type I | MYOT |
| 1.07407833 | 0.595 | 0.173 | 0 | Muscle Type I | PLIN5 |
| 1.07381726 | 0.838 | 0.366 | 0 | Muscle Type I | CKMT2 |
| 1.07273603 | 0.853 | 0.448 | 0 | Muscle Type I | SYNPO2 |
| 1.06564114 | 0.978 | 0.556 | 0 | Muscle Type I | ACTN2 |
| 1.05694305 | 0.515 | 0.072 | 0 | Muscle Type I | MYL3 |
| 1.05498063 | 0.999 | 0.769 | 0 | Muscle Type I | MYBPC1 |
| 1.04299413 | 0.983 | 0.716 | 0 | Muscle Type I | SGCD |
| 1.04055793 | 0.665 | 0.259 | 0 | Muscle Type I | P4HA1 |
| 1.03384749 | 0.299 | 0.052 | 0 | Muscle Type I | NKAIN3 |
| 3.60198318 | 0.731 | 0.135 | 0 | Muscle Type II | MYH1 |
| 3.21586573 | 0.792 | 0.251 | 0 | Muscle Type II | MYH2 |
| 3.17167175 | 0.976 | 0.225 | 0 | Muscle Type II | ATP2A1 |
| 2.87240991 | 0.986 | 0.271 | 0 | Muscle Type II | TNNT3 |
| 2.50938067 | 0.678 | 0.111 | 0 | Muscle Type II | MYHAS |
| 2.50912071 | 0.807 | 0.124 | 0 | Muscle Type II | MYBPC2 |
| 2.32199773 | 0.865 | 0.269 | 0 | Muscle Type II | MYLPF |
| 2.11669669 | 0.81 | 0.304 | 0 | Muscle Type II | MYL1 |
| 1.9888197 | 0.481 | 0.076 | 0 | Muscle Type II | ATRNL1 |
| 1.98266026 | 0.911 | 0.343 | 0 | Muscle Type II | TPM1 |
| 1.91954084 | 0.827 | 0.212 | 0 | Muscle Type II | TNNI2 |
| 1.90299083 | 0.312 | 0.03 | 0 | Muscle Type II | LINC02107 |
| 1.74123873 | 0.623 | 0.239 | 0 | Muscle Type II | MYLK4 |
| 1.70192377 | 0.387 | 0.045 | 0 | Muscle Type II | ACTN3 |
| 1.6404976 | 0.473 | 0.173 | 0 | Muscle Type II | GGT7 |
| 1.57733609 | 0.714 | 0.257 | 0 | Muscle Type II | PFKM |
| 1.56552765 | 0.351 | 0.054 | 0 | Muscle Type II | AC112206.2 |
| 1.51525403 | 0.895 | 0.555 | 0 | Muscle Type II | DENND2C |
| 1.50713964 | 0.87 | 0.497 | 0 | Muscle Type II | RHOBTB1 |
| 1.48628326 | 0.724 | 0.309 | 0 | Muscle Type II | PFKFB1 |
| 1.43498499 | 0.85 | 0.415 | 0 | Muscle Type II | COL4A3 |
| 1.4105992 | 0.681 | 0.27 | 0 | Muscle Type II | ENO3 |
| 1.40906649 | 0.869 | 0.508 | 0 | Muscle Type II | KCNQ5 |
| 1.40472367 | 0.738 | 0.376 | 0 | Muscle Type II | EGF |
| 1.38714312 | 0.541 | 0.224 | 0 | Muscle Type II | MLF1 |
| 1.37756079 | 0.731 | 0.426 | 0 | Muscle Type II | NEDD4 |
| 1.37335496 | 0.995 | 0.797 | 0 | Muscle Type II | FILIP1L |
| 1.36216203 | 0.798 | 0.331 | 0 | Muscle Type II | TNNC2 |
| 1.34668161 | 0.522 | 0.221 | 0 | Muscle Type II | UNC13C |
| 1.34359965 | 0.423 | 0.12 | 0 | Muscle Type II | SH3RF2 |
| 1.33739239 | 0.869 | 0.537 | 0 | Muscle Type II | DEPTOR |
| 1.33352381 | 0.81 | 0.446 | 0 | Muscle Type II | ARHGAP6 |
| 1.31958247 | 0.915 | 0.586 | 0 | Muscle Type II | AGL |
| 1.31586037 | 0.467 | 0.177 | 0 | Muscle Type II | LANCL1-AS1 |
| 1.30458321 | 0.857 | 0.483 | 0 | Muscle Type II | SLC7A2 |
| 1.30236125 | 0.766 | 0.389 | 0 | Muscle Type II | ART3 |
| 1.29063759 | 0.866 | 0.551 | 0 | Muscle Type II | PHTF2 |
| 1.24839231 | 0.985 | 0.674 | 0 | Muscle Type II | PDLIM3 |
| 1.24466598 | 0.958 | 0.697 | 0 | Muscle Type II | SESN1 |
| 1.23530984 | 0.47 | 0.141 | 0 | Muscle Type II | ATP2B2 |
| 1.22344382 | 0.8 | 0.455 | 0 | Muscle Type II | PPP1R3A |
| 1.22304237 | 0.418 | 0.119 | 0 | Muscle Type II | GADL1 |
| 1.2150497 | 0.68 | 0.366 | 0 | Muscle Type II | PGM1 |
| 1.19433888 | 0.731 | 0.431 | 0 | Muscle Type II | NOS1 |
| 1.19332863 | 0.8 | 0.446 | 0 | Muscle Type II | MYOZ1 |
| 1.19293919 | 0.695 | 0.369 | 0 | Muscle Type II | PYGM |
| 1.19129409 | 0.562 | 0.274 | 0 | Muscle Type II | PHKA1 |
| 1.1892849 | 0.502 | 0.197 | 0 | Muscle Type II | PSTPIP2 |
| 1.16639799 | 0.658 | 0.306 | 0 | Muscle Type II | COL4A4 |
| 1.15726439 | 0.832 | 0.491 | 0 | Muscle Type II | AMPD1 |
| 5.36688541 | 0.521 | 0.053 | 0 | FAPs | LRRTM4 |
| 4.83644062 | 0.921 | 0.065 | 0 | FAPs | NEGR1 |
| 4.34885191 | 0.872 | 0.056 | 0 | FAPs | NOVA1 |
| 3.86689131 | 0.89 | 0.056 | 0 | FAPs | DCN |
| 3.73521936 | 0.936 | 0.087 | 0 | FAPs | EBF1 |
| 3.58523186 | 0.785 | 0.081 | 0 | FAPs | FBN1 |
| 3.4850202 | 0.616 | 0.054 | 0 | FAPs | COL15A1 |
| 3.46181543 | 0.416 | 0.026 | 0 | FAPs | SCN7A |
| 3.44216934 | 0.79 | 0.044 | 0 | FAPs | TNXB |
| 3.42135385 | 0.506 | 0.033 | 0 | FAPs | ROBO2 |
| 3.37311396 | 0.774 | 0.03 | 0 | FAPs | COL6A3 |
| 3.35794196 | 0.825 | 0.07 | 0 | FAPs | ABCA8 |
| 3.34843863 | 0.8 | 0.06 | 0 | FAPs | DCLK1 |
| 3.29192007 | 0.947 | 0.501 | 0 | FAPs | LAMA2 |
| 3.15078771 | 0.289 | 0.015 | 0 | FAPs | PDZRN4 |
| 3.12898395 | 0.763 | 0.067 | 0 | FAPs | PID1 |
| 3.07833665 | 0.781 | 0.031 | 0 | FAPs | EBF2 |
| 3.02072866 | 0.924 | 0.08 | 0 | FAPs | DLC1 |
| 2.95992138 | 0.644 | 0.106 | 0 | FAPs | KAZN |
| 2.88142565 | 0.649 | 0.077 | 0 | FAPs | SMOC2 |
| 2.81845929 | 0.514 | 0.029 | 0 | FAPs | KCND2 |
| 2.80881542 | 0.527 | 0.018 | 0 | FAPs | BMPER |
| 2.77185066 | 0.72 | 0.032 | 0 | FAPs | COL1A2 |
| 2.73227234 | 0.774 | 0.165 | 0 | FAPs | ABCA9 |
| 2.70699639 | 0.833 | 0.09 | 0 | FAPs | SOX5 |
| 2.65956578 | 0.713 | 0.029 | 0 | FAPs | VIT |
| 2.63357625 | 0.731 | 0.405 | 0 | FAPs | ABCA10 |
| 2.5733929 | 0.413 | 0.014 | 0 | FAPs | ADH1B |
| 2.57212057 | 0.52 | 0.115 | 0 | FAPs | SDK1 |
| 2.52690428 | 0.865 | 0.249 | 0 | FAPs | EGFR |
| 2.5186937 | 0.587 | 0.02 | 0 | FAPs | SVEP1 |
| 2.51710094 | 0.585 | 0.035 | 0 | FAPs | ADAMTSL3 |
| 2.50378848 | 0.491 | 0.081 | 0 | FAPs | MME |
| 2.47671695 | 0.382 | 0.021 | 0 | FAPs | NCAM2 |
| 2.45990423 | 0.521 | 0.012 | 0 | FAPs | MFAP5 |
| 2.43312014 | 0.741 | 0.107 | 0 | FAPs | HSPG2 |
| 2.42445823 | 0.627 | 0.096 | 0 | FAPs | CAMK1D |
| 2.42362522 | 0.422 | 0.011 | 0 | FAPs | NOX4 |
| 2.41113172 | 0.783 | 0.109 | 0 | FAPs | FBXL7 |
| 2.38482307 | 0.763 | 0.204 | 0 | FAPs | ABCA6 |
| 2.37799344 | 0.533 | 0.015 | 0 | FAPs | ITGA11 |
| 2.37628388 | 0.612 | 0.045 | 0 | FAPs | COL3A1 |
| 2.37448776 | 0.465 | 0.028 | 0 | FAPs | CCDC80 |
| 2.36976996 | 0.706 | 0.14 | 0 | FAPs | GSN |
| 2.32844281 | 0.643 | 0.08 | 0 | FAPs | UST |
| 2.32571923 | 0.618 | 0.076 | 0 | FAPs | GRK5 |
| 2.30475157 | 0.499 | 0.013 | 0 | FAPs | COL12A1 |
| 2.2938846 | 0.548 | 0.057 | 0 | FAPs | COL4A2 |
| 2.29132694 | 0.691 | 0.155 | 0 | FAPs | ABI3BP |
| 2.2899734 | 0.744 | 0.172 | 0 | FAPs | RUNX1T1 |
| 4.75250915 | 0.732 | 0.007 | 0 | Satellite Cells | AC096577.1 |
| 4.06508908 | 0.822 | 0.022 | 0 | Satellite Cells | PAX7 |
| 3.88753278 | 0.691 | 0.01 | 0 | Satellite Cells | CALCR |
| 3.79689447 | 0.765 | 0.137 | 0 | Satellite Cells | DIRC3 |
| 3.7508206 | 0.969 | 0.148 | 0 | Satellite Cells | MEG3 |
| 3.53892157 | 0.659 | 0.024 | 0 | Satellite Cells | TRHDE |
| 3.44319208 | 0.658 | 0.045 | 0 | Satellite Cells | CDH4 |
| 3.40336322 | 0.991 | 0.581 | 0 | Satellite Cells | CADM2 |
| 3.35092475 | 0.758 | 0.076 | 0 | Satellite Cells | GPC6 |
| 3.35041416 | 0.835 | 0.161 | 0 | Satellite Cells | CLCN5 |
| 3.28729208 | 0.77 | 0.091 | 0 | Satellite Cells | 01-Mar |
| 3.27910181 | 0.905 | 0.127 | 0 | Satellite Cells | HMCN2 |
| 3.25761115 | 0.536 | 0.013 | 0 | Satellite Cells | AC004053.1 |
| 3.24755219 | 0.802 | 0.099 | 0 | Satellite Cells | MEG8 |
| 3.1335308 | 0.709 | 0.043 | 0 | Satellite Cells | TENM4 |
| 2.70266598 | 0.571 | 0.04 | 0 | Satellite Cells | CNKSR3 |
| 2.69391644 | 0.742 | 0.188 | 0 | Satellite Cells | FRMD4A |
| 2.67354165 | 0.485 | 0.018 | 0 | Satellite Cells | TMEFF2 |
| 2.64252394 | 0.608 | 0.158 | 0 | Satellite Cells | KCNQ1OT1 |
| 2.63473516 | 0.727 | 0.09 | 0 | Satellite Cells | NCAM1 |
| 2.60430584 | 0.769 | 0.152 | 0 | Satellite Cells | DOCK9 |
| 2.5961996 | 0.428 | 0.005 | 0 | Satellite Cells | NLGN4X |
| 2.58635704 | 0.593 | 0.048 | 0 | Satellite Cells | DANT2 |
| 2.58233769 | 0.768 | 0.295 | 0 | Satellite Cells | KANK1 |
| 2.50430604 | 0.6 | 0.072 | 0 | Satellite Cells | MSC-AS1 |
| 2.47745033 | 0.79 | 0.318 | 0 | Satellite Cells | SPATS2L |
| 2.45427565 | 0.87 | 0.418 | 0 | Satellite Cells | TLN2 |
| 2.44271633 | 0.718 | 0.228 | 0 | Satellite Cells | PTCHD1-AS |
| 2.43261188 | 0.458 | 0.028 | 0 | Satellite Cells | GNA14 |
| 2.41831303 | 0.559 | 0.081 | 0 | Satellite Cells | NTN4 |
| 2.40407071 | 0.59 | 0.152 | 0 | Satellite Cells | DYNC1I1 |
| 2.33071535 | 0.604 | 0.129 | 0 | Satellite Cells | MEGF10 |
| 2.3046954 | 0.569 | 0.073 | 0 | Satellite Cells | PON2 |
| 2.30284589 | 0.509 | 0.033 | 0 | Satellite Cells | CHN1 |
| 2.26311237 | 0.51 | 0.063 | 0 | Satellite Cells | ITGBL1 |
| 2.24443552 | 0.703 | 0.247 | 0 | Satellite Cells | ADAMTS9-AS2 |
| 2.20122145 | 0.569 | 0.125 | 0 | Satellite Cells | PRKD1 |
| 2.19824795 | 0.64 | 0.124 | 0 | Satellite Cells | SPARCL1 |
| 2.19242704 | 0.518 | 0.086 | 0 | Satellite Cells | FN1 |
| 2.15291889 | 0.448 | 0.022 | 0 | Satellite Cells | OLFML2B |
| 2.14288412 | 0.88 | 0.339 | 0 | Satellite Cells | PLXDC2 |
| 2.109816 | 0.564 | 0.106 | 0 | Satellite Cells | RASSF4 |
| 2.0858242 | 0.487 | 0.041 | 0 | Satellite Cells | PXDN |
| 2.08342895 | 0.812 | 0.457 | 0 | Satellite Cells | PPP1R9A |
| 2.06002375 | 0.398 | 0.061 | 0 | Satellite Cells | LINC01239 |
| 2.02717126 | 0.373 | 0.007 | 0 | Satellite Cells | GRIK4 |
| 2.02122612 | 0.316 | 0.007 | 0 | Satellite Cells | CTNND2 |
| 2.01843641 | 0.619 | 0.135 | 0 | Satellite Cells | NAV1 |
| 2.01474014 | 0.426 | 0.045 | 0 | Satellite Cells | MUSK |
| 1.98450862 | 0.56 | 0.15 | 0 | Satellite Cells | MDFIC |
| 4.8822424 | 0.853 | 0.016 | 0 | Macrophages | F13A1 |
| 4.86871583 | 0.952 | 0.064 | 0 | Macrophages | FRMD4B |
| 4.46071449 | 0.947 | 0.452 | 0 | Macrophages | RBPJ |
| 4.26586887 | 0.872 | 0.017 | 0 | Macrophages | MRC1 |
| 3.83040802 | 0.787 | 0.019 | 0 | Macrophages | P2RY14 |
| 3.75775064 | 0.717 | 0.067 | 0 | Macrophages | COLEC12 |
| 3.68431779 | 0.797 | 0.069 | 0 | Macrophages | RGL1 |
| 3.62584249 | 0.851 | 0.026 | 0 | Macrophages | IQGAP2 |
| 3.62071212 | 0.795 | 0.146 | 0 | Macrophages | MAMDC2 |
| 3.60471483 | 0.821 | 0.014 | 0 | Macrophages | RBM47 |
| 3.55490999 | 0.789 | 0.197 | 0 | Macrophages | NAV2 |
| 3.47875888 | 0.678 | 0.07 | 0 | Macrophages | LGMN |
| 3.38074967 | 0.917 | 0.217 | 0 | Macrophages | SLC9A9 |
| 3.36657104 | 0.774 | 0.094 | 0 | Macrophages | MAN1A1 |
| 3.34909137 | 0.776 | 0.007 | 0 | Macrophages | MS4A6A |
| 3.33987856 | 0.751 | 0.029 | 0 | Macrophages | SCN9A |
| 3.26189938 | 0.749 | 0.061 | 0 | Macrophages | SLC8A1 |
| 3.25047661 | 0.722 | 0.009 | 0 | Macrophages | MS4A4E |
| 3.21594475 | 0.791 | 0.014 | 0 | Macrophages | TBXAS1 |
| 3.19281696 | 0.708 | 0.039 | 0 | Macrophages | DAB2 |
| 3.17841979 | 0.689 | 0.04 | 0 | Macrophages | HRH1 |
| 3.16629388 | 0.934 | 0.167 | 0 | Macrophages | LRMDA |
| 3.11522617 | 0.65 | 0.015 | 0 | Macrophages | LYVE1 |
| 3.06096613 | 0.79 | 0.027 | 0 | Macrophages | DOCK2 |
| 2.97431022 | 0.737 | 0.007 | 0 | Macrophages | SYK |
| 2.95085084 | 0.7 | 0.113 | 0 | Macrophages | SELENOP |
| 2.94480637 | 0.648 | 0.01 | 0 | Macrophages | STAB1 |
| 2.90940678 | 0.718 | 0.016 | 0 | Macrophages | ATP8B4 |
| 2.89124177 | 0.582 | 0.023 | 0 | Macrophages | RTN1 |
| 2.85547687 | 0.758 | 0.202 | 0 | Macrophages | PDGFC |
| 2.83220383 | 0.779 | 0.189 | 0 | Macrophages | NRP1 |
| 2.82996286 | 0.465 | 0.023 | 0 | Macrophages | LSAMP |
| 2.82590147 | 0.893 | 0.316 | 0 | Macrophages | ZEB2 |
| 2.73740569 | 0.662 | 0.026 | 0 | Macrophages | ADAP2 |
| 2.73509534 | 0.576 | 0.006 | 0 | Macrophages | CD163L1 |
| 2.70672425 | 0.751 | 0.266 | 0 | Macrophages | ITSN1 |
| 2.70374774 | 0.611 | 0.004 | 0 | Macrophages | LILRB5 |
| 2.673384 | 0.67 | 0.01 | 0 | Macrophages | CSF1R |
| 2.66126764 | 0.573 | 0.006 | 0 | Macrophages | SIGLEC1 |
| 2.63650113 | 0.58 | 0.026 | 0 | Macrophages | CPM |
| 2.62361991 | 0.647 | 0.014 | 0 | Macrophages | EMB |
| 2.60499806 | 0.72 | 0.1 | 0 | Macrophages | DAPK1 |
| 2.6040385 | 0.628 | 0.028 | 0 | Macrophages | SLCO2B1 |
| 2.59535616 | 0.778 | 0.287 | 0 | Macrophages | STARD13 |
| 2.58017583 | 0.627 | 0.093 | 0 | Macrophages | DNM1 |
| 2.57902682 | 0.605 | 0.004 | 0 | Macrophages | CD163 |
| 2.57280267 | 0.659 | 0.105 | 0 | Macrophages | AFF3 |
| 2.55051511 | 0.83 | 0.251 | 0 | Macrophages | ZSWIM6 |
| 2.52922507 | 0.686 | 0.104 | 0 | Macrophages | TNFAIP2 |
| 2.52819495 | 0.759 | 0.059 | 0 | Macrophages | FLI1 |
| 2.70701584 | 0.366 | 0.016 | 0 | Differentiating Muscle | COL19A1 |
| 2.13840535 | 0.298 | 0.033 | 0 | Differentiating Muscle | DNAH11 |
| 2.11234746 | 0.457 | 0.098 | 0 | Differentiating Muscle | NCAM1 |
| 2.08463021 | 0.408 | 0.198 | 1.53E-189 | Differentiating Muscle | LRRK2 |
| 1.84459552 | 0.316 | 0.048 | 0 | Differentiating Muscle | GALNT17 |
| 1.69807374 | 0.511 | 0.2 | 0 | Differentiating Muscle | ARHGAP28 |
| 1.63788677 | 0.503 | 0.204 | 0 | Differentiating Muscle | MDM2 |
| 1.63161351 | 0.261 | 0.08 | 8.34E-241 | Differentiating Muscle | COL21A1 |
| 1.49338812 | 0.338 | 0.099 | 0 | Differentiating Muscle | EFCAB7 |
| 1.48263946 | 0.386 | 0.174 | 1.03E-186 | Differentiating Muscle | RUNX1 |
| 1.46524838 | 0.515 | 0.241 | 2.14E-273 | Differentiating Muscle | ASTN2 |
| 1.4417495 | 0.416 | 0.149 | 0 | Differentiating Muscle | CASQ2 |
| 1.42796616 | 0.411 | 0.204 | 8.28E-158 | Differentiating Muscle | RNLS |
| 1.42142726 | 0.752 | 0.45 | 9.78E-287 | Differentiating Muscle | ARPP21 |
| 1.40425948 | 0.406 | 0.164 | 5.80E-265 | Differentiating Muscle | FAM184B |
| 1.36498797 | 0.755 | 0.49 | 1.44E-250 | Differentiating Muscle | OSBPL6 |
| 1.31904789 | 0.606 | 0.329 | 6.14E-261 | Differentiating Muscle | SLC7A6 |
| 1.30749605 | 0.332 | 0.139 | 3.96E-176 | Differentiating Muscle | AF165147.1 |
| 1.26888876 | 0.321 | 0.096 | 0 | Differentiating Muscle | PPP1R14C |
| 1.26731771 | 0.392 | 0.137 | 0 | Differentiating Muscle | ADAM23 |
| 1.26347321 | 0.358 | 0.154 | 3.59E-194 | Differentiating Muscle | FAM13C |
| 1.24436218 | 0.31 | 0.107 | 7.88E-237 | Differentiating Muscle | PLCE1 |
| 1.21309058 | 0.265 | 0.183 | 1.26E-23 | Differentiating Muscle | AL390957.1 |
| 1.20652115 | 0.96 | 0.734 | 0 | Differentiating Muscle | RBFOX1 |
| 1.19638069 | 0.682 | 0.383 | 1.56E-270 | Differentiating Muscle | BEST3 |
| 1.16740399 | 0.407 | 0.149 | 3.05E-285 | Differentiating Muscle | KCNN3 |
| 1.14903994 | 0.904 | 0.606 | 0 | Differentiating Muscle | TP63 |
| 1.13325873 | 0.802 | 0.622 | 3.90E-131 | Differentiating Muscle | XIRP2 |
| 1.12156099 | 0.405 | 0.151 | 2.00E-278 | Differentiating Muscle | FREM2 |
| 1.12118145 | 0.252 | 0.108 | 2.40E-121 | Differentiating Muscle | PRUNE2 |
| 1.10997857 | 0.618 | 0.425 | 1.80E-120 | Differentiating Muscle | GSE1 |
| 1.09326733 | 0.533 | 0.264 | 2.22E-231 | Differentiating Muscle | EPHB1 |
| 1.08022853 | 0.365 | 0.142 | 1.10E-232 | Differentiating Muscle | CCDC39.1 |
| 1.07553143 | 0.411 | 0.244 | 2.60E-98 | Differentiating Muscle | SH3PXD2A |
| 1.07201566 | 0.623 | 0.366 | 2.90E-187 | Differentiating Muscle | FLRT2 |
| 1.06395708 | 0.365 | 0.147 | 1.44E-208 | Differentiating Muscle | TMEM178B |
| 1.0631268 | 0.441 | 0.204 | 2.46E-211 | Differentiating Muscle | AMOTL1 |
| 1.03866605 | 0.616 | 0.358 | 1.83E-183 | Differentiating Muscle | SLC24A3 |
| 1.0275498 | 0.609 | 0.364 | 5.57E-172 | Differentiating Muscle | INPP4B |
| 1.00174695 | 0.491 | 0.291 | 1.35E-123 | Differentiating Muscle | ADARB1 |
| 1.00016953 | 0.325 | 0.099 | 1.59E-296 | Differentiating Muscle | CDC42EP3 |
| 0.99604536 | 0.538 | 0.378 | 1.92E-65 | Differentiating Muscle | LINC01091 |
| 0.98841721 | 0.391 | 0.179 | 1.99E-178 | Differentiating Muscle | RRAD |
| 0.94960273 | 0.613 | 0.378 | 3.64E-154 | Differentiating Muscle | PLA2G4C |
| 0.94745797 | 0.542 | 0.293 | 4.86E-189 | Differentiating Muscle | TRIM55 |
| 0.93980259 | 0.745 | 0.6 | 1.33E-84 | Differentiating Muscle | FOXO1 |
| 0.93886382 | 0.255 | 0.101 | 1.24E-140 | Differentiating Muscle | COBLL1 |
| 0.92835866 | 0.289 | 0.117 | 1.89E-156 | Differentiating Muscle | ITGA9 |
| 0.91477933 | 0.31 | 0.105 | 2.17E-237 | Differentiating Muscle | UCK2 |
| 0.90518116 | 0.607 | 0.403 | 3.75E-120 | Differentiating Muscle | TULP4 |
| 4.87576309 | 0.918 | 0.012 | 0 | Endothelial | MECOM |
| 4.35006148 | 0.945 | 0.072 | 0 | Endothelial | LDB2 |
| 4.08820924 | 0.882 | 0.01 | 0 | Endothelial | PTPRB |
| 4.03070028 | 0.879 | 0.016 | 0 | Endothelial | EMCN |
| 4.02243057 | 0.828 | 0.006 | 0 | Endothelial | ANO2 |
| 3.79025025 | 0.734 | 0.015 | 0 | Endothelial | SNTG2 |
| 3.73721442 | 0.822 | 0.011 | 0 | Endothelial | VWF |
| 3.68151574 | 0.754 | 0.03 | 0 | Endothelial | ST6GALNAC3 |
| 3.57157342 | 0.734 | 0.012 | 0 | Endothelial | FLT1 |
| 3.51167498 | 0.821 | 0.01 | 0 | Endothelial | EGFL7 |
| 3.51098134 | 0.827 | 0.038 | 0 | Endothelial | PECAM1 |
| 3.49281801 | 0.651 | 0.007 | 0 | Endothelial | TPO |
| 3.43093673 | 0.747 | 0.008 | 0 | Endothelial | CYYR1 |
| 3.34910781 | 0.776 | 0.24 | 0 | Endothelial | ARL15 |
| 3.2537259 | 0.749 | 0.062 | 0 | Endothelial | MCTP1 |
| 3.13325884 | 0.457 | 0.003 | 0 | Endothelial | BTNL9 |
| 3.03468636 | 0.343 | 0.011 | 0 | Endothelial | FAM155A |
| 3.03384572 | 0.821 | 0.073 | 0 | Endothelial | FLI1 |
| 3.01888844 | 0.797 | 0.147 | 0 | Endothelial | PLCB4 |
| 3.00033135 | 0.754 | 0.013 | 0 | Endothelial | ERG |
| 2.98713072 | 0.722 | 0.109 | 0 | Endothelial | PKP4 |
| 2.98290598 | 0.775 | 0.105 | 0 | Endothelial | PITPNC1 |
| 2.9715622 | 0.702 | 0.058 | 0 | Endothelial | ITGA6 |
| 2.9267261 | 0.72 | 0.014 | 0 | Endothelial | SHANK3 |
| 2.92326169 | 0.639 | 0.003 | 0 | Endothelial | ADGRL4 |
| 2.89321145 | 0.564 | 0.04 | 0 | Endothelial | CCDC85A |
| 2.87173446 | 0.744 | 0.133 | 0 | Endothelial | TSHZ2 |
| 2.86491623 | 0.649 | 0.006 | 0 | Endothelial | CXorf36 |
| 2.85254857 | 0.53 | 0.016 | 0 | Endothelial | DACH1 |
| 2.84795505 | 0.729 | 0.078 | 0 | Endothelial | STOX2 |
| 2.8434149 | 0.612 | 0.016 | 0 | Endothelial | RASGRF2 |
| 2.83893952 | 0.756 | 0.049 | 0 | Endothelial | ARHGAP31 |
| 2.83608602 | 0.519 | 0.042 | 0 | Endothelial | THSD7A |
| 2.78948529 | 0.684 | 0.052 | 0 | Endothelial | EPAS1 |
| 2.75940312 | 0.76 | 0.09 | 0 | Endothelial | PREX2 |
| 2.75878536 | 0.651 | 0.033 | 0 | Endothelial | PLEKHG1 |
| 2.71208827 | 0.983 | 0.734 | 0 | Endothelial | PTPRM |
| 2.70980362 | 0.56 | 0.116 | 0 | Endothelial | MYRIP |
| 2.69967462 | 0.669 | 0.053 | 0 | Endothelial | CSGALNACT1 |
| 2.6932681 | 0.408 | 0.014 | 0 | Endothelial | TLL1 |
| 2.6802064 | 0.551 | 0.066 | 0 | Endothelial | ABLIM3 |
| 2.64032044 | 0.679 | 0.178 | 0 | Endothelial | ZNF385D |
| 2.63725015 | 0.928 | 0.184 | 0 | Endothelial | LRMDA |
| 2.62710733 | 0.484 | 0.018 | 0 | Endothelial | ADGRF5 |
| 2.61122055 | 0.594 | 0.095 | 0 | Endothelial | MGLL |
| 2.6065919 | 0.892 | 0.173 | 0 | Endothelial | ELMO1 |
| 2.54376369 | 0.607 | 0.054 | 0 | Endothelial | GALNT18 |
| 2.51693361 | 0.585 | 0.059 | 0 | Endothelial | ENG |
| 2.49692911 | 0.506 | 0.038 | 0 | Endothelial | TMTC2 |
| 2.48158605 | 0.634 | 0.039 | 0 | Endothelial | FLNB |
| 4.44942402 | 0.915 | 0.01 | 0 | Smooth Muscle | CARMN |
| 4.33505611 | 0.95 | 0.073 | 0 | Smooth Muscle | CACNA1C |
| 4.25417169 | 0.817 | 0.039 | 0 | Smooth Muscle | GUCY1A2 |
| 4.18354928 | 0.789 | 0.059 | 0 | Smooth Muscle | EGFLAM |
| 4.00595646 | 0.962 | 0.184 | 0 | Smooth Muscle | DLC1 |
| 3.97619613 | 0.823 | 0.143 | 0 | Smooth Muscle | FRMD3 |
| 3.96928847 | 0.882 | 0.145 | 0 | Smooth Muscle | EPS8 |
| 3.61232024 | 0.863 | 0.063 | 0 | Smooth Muscle | PDGFRB |
| 3.60854355 | 0.924 | 0.147 | 0 | Smooth Muscle | NR2F2-AS1 |
| 3.57715006 | 0.74 | 0.172 | 0 | Smooth Muscle | PDZD2 |
| 3.48463003 | 0.76 | 0.099 | 0 | Smooth Muscle | AC012409.2 |
| 3.46687074 | 0.736 | 0.026 | 0 | Smooth Muscle | MYO1B |
| 3.44600111 | 0.836 | 0.089 | 0 | Smooth Muscle | RBPMS |
| 3.43181672 | 0.453 | 0.018 | 0 | Smooth Muscle | RGS6 |
| 3.42538949 | 0.664 | 0.019 | 0 | Smooth Muscle | RGS5 |
| 3.35267237 | 0.862 | 0.065 | 0 | Smooth Muscle | CLMN |
| 3.27194522 | 0.866 | 0.133 | 0 | Smooth Muscle | LHFPL6 |
| 3.22951613 | 0.963 | 0.283 | 0 | Smooth Muscle | CALD1 |
| 3.22784396 | 0.832 | 0.081 | 0 | Smooth Muscle | IGFBP7 |
| 3.09571113 | 0.558 | 0.03 | 0 | Smooth Muscle | PDE1C |
| 3.07129739 | 0.399 | 0.046 | 0 | Smooth Muscle | KCNAB1 |
| 3.06845726 | 0.682 | 0.074 | 0 | Smooth Muscle | PDE3A |
| 2.95817767 | 0.673 | 0.024 | 0 | Smooth Muscle | GUCY1A1 |
| 2.89523852 | 0.92 | 0.18 | 0 | Smooth Muscle | SOX5 |
| 2.84607813 | 0.551 | 0.024 | 0 | Smooth Muscle | ACTA2 |
| 2.84212635 | 0.687 | 0.022 | 0 | Smooth Muscle | NOTCH3 |
| 2.80804615 | 0.608 | 0.046 | 0 | Smooth Muscle | RIPOR3 |
| 2.76021099 | 0.863 | 0.26 | 0 | Smooth Muscle | RASAL2 |
| 2.75261641 | 0.597 | 0.062 | 0 | Smooth Muscle | CACNB2 |
| 2.74139594 | 0.502 | 0.024 | 0 | Smooth Muscle | AL356258.1 |
| 2.72717823 | 0.825 | 0.26 | 0 | Smooth Muscle | ADAMTS9-AS2 |
| 2.72356067 | 0.632 | 0.005 | 0 | Smooth Muscle | MRVI1 |
| 2.69416206 | 0.712 | 0.054 | 0 | Smooth Muscle | SPECC1 |
| 2.69086646 | 0.47 | 0.009 | 0 | Smooth Muscle | SLC16A12 |
| 2.66090378 | 0.549 | 0.001 | 0 | Smooth Muscle | FHL5 |
| 2.65191605 | 0.261 | 0.035 | 1.34E-231 | Smooth Muscle | MYH11 |
| 2.6278076 | 0.574 | 0.009 | 0 | Smooth Muscle | NFASC |
| 2.54897913 | 0.575 | 0.061 | 0 | Smooth Muscle | ADGRB3 |
| 2.53723162 | 0.399 | 0.007 | 0 | Smooth Muscle | AL499616.1 |
| 2.51667432 | 0.755 | 0.138 | 0 | Smooth Muscle | SPARCL1 |
| 2.49797483 | 0.562 | 0.068 | 0 | Smooth Muscle | ADAMTS12 |
| 2.48569963 | 0.413 | 0.058 | 0 | Smooth Muscle | SORBS2 |
| 2.46604675 | 0.729 | 0.243 | 0 | Smooth Muscle | SH3RF1 |
| 2.46517177 | 0.732 | 0.153 | 0 | Smooth Muscle | PLCB4 |
| 2.43854653 | 0.811 | 0.193 | 0 | Smooth Muscle | MAML2 |
| 2.43746213 | 0.853 | 0.371 | 0 | Smooth Muscle | INPP4B |
| 2.43373641 | 0.625 | 0.058 | 0 | Smooth Muscle | BMP5 |
| 2.43200493 | 0.537 | 0.035 | 0 | Smooth Muscle | COL5A3 |
| 2.42687747 | 0.421 | 0.085 | 1.47E-236 | Smooth Muscle | SLIT3 |
| 2.42023881 | 0.483 | 0.028 | 0 | Smooth Muscle | SEMA5A |
| 4.58539783 | 0.944 | 0.062 | 0 | Immune Cells | ARHGAP15 |
| 4.39345782 | 0.911 | 0.014 | 0 | Immune Cells | SKAP1 |
| 3.94825478 | 0.881 | 0.039 | 0 | Immune Cells | PTPRC |
| 3.88885682 | 0.695 | 0.002 | 0 | Immune Cells | THEMIS |
| 3.60609461 | 0.814 | 0.025 | 0 | Immune Cells | IKZF1 |
| 3.49502095 | 0.835 | 0.156 | 0 | Immune Cells | RIPOR2 |
| 3.44924432 | 0.45 | 0.031 | 0 | Immune Cells | C15orf53 |
| 3.43060692 | 0.916 | 0.172 | 0 | Immune Cells | ANKRD44 |
| 3.40560396 | 0.82 | 0.068 | 0 | Immune Cells | PARP8 |
| 3.37477488 | 0.723 | 0.006 | 0 | Immune Cells | SLFN12L |
| 3.31602016 | 0.506 | 0.012 | 0 | Immune Cells | TOX |
| 3.2557841 | 0.694 | 0.001 | 0 | Immune Cells | BCL11B |
| 3.25284525 | 0.659 | 0.01 | 0 | Immune Cells | ITGA4 |
| 3.2003431 | 0.545 | 0.025 | 0 | Immune Cells | PCAT1 |
| 3.18915584 | 0.751 | 0.03 | 0 | Immune Cells | FYB1 |
| 3.14569407 | 0.511 | 0.005 | 0 | Immune Cells | LINC01934 |
| 3.08792224 | 0.812 | 0.101 | 0 | Immune Cells | CCND3 |
| 3.0680975 | 0.827 | 0.079 | 0 | Immune Cells | DOCK8 |
| 3.05952162 | 0.596 | 0.01 | 0 | Immune Cells | SAMD3 |
| 2.94460761 | 0.666 | 0.071 | 0 | Immune Cells | ETS1 |
| 2.92154486 | 0.605 | 0.022 | 0 | Immune Cells | CD247 |
| 2.91790315 | 0.728 | 0.084 | 0 | Immune Cells | CHST11 |
| 2.90634838 | 0.728 | 0.105 | 0 | Immune Cells | PIP4K2A |
| 2.8717821 | 0.593 | 0.004 | 0 | Immune Cells | CARD11 |
| 2.86646911 | 0.684 | 0.031 | 0 | Immune Cells | CD96 |
| 2.78930281 | 0.639 | 0.048 | 0 | Immune Cells | PCED1B |
| 2.78048856 | 0.652 | 0.055 | 0 | Immune Cells | TC2N |
| 2.73372717 | 0.613 | 0.039 | 0 | Immune Cells | TNFAIP8 |
| 2.73230374 | 0.682 | 0.046 | 0 | Immune Cells | APBB1IP |
| 2.72207928 | 0.58 | 0.007 | 0 | Immune Cells | STAT4 |
| 2.67569812 | 0.555 | 0.001 | 0 | Immune Cells | CD2 |
| 2.66425188 | 0.54 | 0.003 | 0 | Immune Cells | ITK |
| 2.64950099 | 0.85 | 0.373 | 9.18E-268 | Immune Cells | INPP4B |
| 2.64763958 | 0.481 | 0.005 | 0 | Immune Cells | CAMK4 |
| 2.64199758 | 0.715 | 0.053 | 0 | Immune Cells | DOCK2 |
| 2.61098457 | 0.42 | 0.001 | 0 | Immune Cells | IL7R |
| 2.60772734 | 0.758 | 0.148 | 0 | Immune Cells | DOCK10 |
| 2.57844833 | 0.73 | 0.055 | 0 | Immune Cells | IQGAP2 |
| 2.55996946 | 0.705 | 0.113 | 0 | Immune Cells | PITPNC1 |
| 2.51010371 | 0.507 | 0.005 | 0 | Immune Cells | GRAP2 |
| 2.49834604 | 0.669 | 0.153 | 0 | Immune Cells | KIAA1551 |
| 2.49470885 | 0.583 | 0.035 | 0 | Immune Cells | MYO1F |
| 2.47176978 | 0.657 | 0.113 | 0 | Immune Cells | CD44 |
| 2.45556258 | 0.519 | 0.01 | 0 | Immune Cells | ITGAL |
| 2.4476903 | 0.779 | 0.179 | 0 | Immune Cells | RUNX1 |
| 2.43986702 | 0.728 | 0.137 | 0 | Immune Cells | FYN |
| 2.42326069 | 0.534 | 0.018 | 0 | Immune Cells | CCDC88C |
| 2.42285417 | 0.652 | 0.163 | 1.03E-281 | Immune Cells | MDFIC |
| 2.40896858 | 0.39 | 0.012 | 0 | Immune Cells | PPP2R2B |
| 2.40432442 | 0.537 | 0.03 | 0 | Immune Cells | PIK3R5 |
| 4.99242496 | 0.967 | 0.005 | 0 | Lymphatic Endothelial | PKHD1L1 |
| 4.52347936 | 0.979 | 0.087 | 0 | Lymphatic Endothelial | STOX2 |
| 4.2955808 | 0.869 | 0.003 | 0 | Lymphatic Endothelial | MMRN1 |
| 4.27070546 | 0.893 | 0.041 | 0 | Lymphatic Endothelial | ST6GALNAC3 |
| 4.24993682 | 0.8 | 0.009 | 0 | Lymphatic Endothelial | NRG3 |
| 4.23882344 | 0.943 | 0.1 | 0 | Lymphatic Endothelial | EFNA5 |
| 4.20372742 | 0.618 | 0.024 | 0 | Lymphatic Endothelial | AL357507.1 |
| 3.98515134 | 0.976 | 0.289 | 0 | Lymphatic Endothelial | PPFIBP1 |
| 3.88668032 | 0.734 | 0.009 | 0 | Lymphatic Endothelial | RELN |
| 3.5979962 | 0.731 | 0.008 | 0 | Lymphatic Endothelial | LINC02147 |
| 3.51750996 | 0.943 | 0.079 | 0 | Lymphatic Endothelial | PTPRE |
| 3.49966655 | 0.842 | 0.036 | 0 | Lymphatic Endothelial | NRP2 |
| 3.46421602 | 0.773 | 0.024 | 0 | Lymphatic Endothelial | PIEZO2 |
| 3.43037499 | 0.857 | 0.027 | 0 | Lymphatic Endothelial | SNTG2 |
| 3.42421188 | 0.91 | 0.118 | 0 | Lymphatic Endothelial | DOCK5 |
| 3.38201191 | 0.94 | 0.257 | 1.12E-283 | Lymphatic Endothelial | TFPI |
| 3.29114822 | 0.728 | 0.066 | 0 | Lymphatic Endothelial | GPM6A |
| 3.26185586 | 0.851 | 0.113 | 0 | Lymphatic Endothelial | PDE1A |
| 3.24128744 | 0.94 | 0.141 | 0 | Lymphatic Endothelial | TSHZ2 |
| 3.22183487 | 0.854 | 0.078 | 0 | Lymphatic Endothelial | RHOJ |
| 3.1230221 | 0.86 | 0.122 | 0 | Lymphatic Endothelial | ITGA9 |
| 3.11646232 | 0.785 | 0.064 | 0 | Lymphatic Endothelial | TSPAN5 |
| 3.09267968 | 0.693 | 0.044 | 0 | Lymphatic Endothelial | NTN1 |
| 3.08824138 | 0.648 | 0.012 | 0 | Lymphatic Endothelial | KLHL4 |
| 3.06246534 | 0.866 | 0.087 | 0 | Lymphatic Endothelial | LDB2 |
| 3.0451534 | 0.761 | 0.063 | 0 | Lymphatic Endothelial | CSGALNACT1 |
| 3.04203724 | 0.854 | 0.062 | 0 | Lymphatic Endothelial | VAV3 |
| 3.02695427 | 0.878 | 0.282 | 8.24E-217 | Lymphatic Endothelial | MPP7 |
| 3.01893033 | 0.579 | 0.015 | 0 | Lymphatic Endothelial | AC008691.1 |
| 3.01610349 | 0.761 | 0.017 | 0 | Lymphatic Endothelial | PARD6G |
| 2.95805784 | 0.824 | 0.067 | 0 | Lymphatic Endothelial | ELK3 |
| 2.94826253 | 0.863 | 0.28 | 2.44E-202 | Lymphatic Endothelial | PGM5 |
| 2.93784994 | 0.767 | 0.09 | 0 | Lymphatic Endothelial | PROX1 |
| 2.91424754 | 0.878 | 0.151 | 0 | Lymphatic Endothelial | KIAA1671 |
| 2.86462199 | 0.779 | 0.085 | 0 | Lymphatic Endothelial | HMCN1 |
| 2.85990005 | 0.815 | 0.176 | 4.75E-271 | Lymphatic Endothelial | SMAD1 |
| 2.85561125 | 0.916 | 0.434 | 1.06E-179 | Lymphatic Endothelial | KALRN |
| 2.85183049 | 0.642 | 0.01 | 0 | Lymphatic Endothelial | STAB2 |
| 2.82021757 | 0.436 | 0.016 | 0 | Lymphatic Endothelial | FAM155A |
| 2.81408621 | 0.967 | 0.484 | 5.51E-192 | Lymphatic Endothelial | MAGI1 |
| 2.81257851 | 0.752 | 0.07 | 0 | Lymphatic Endothelial | STK32B |
| 2.796392 | 0.904 | 0.242 | 3.71E-254 | Lymphatic Endothelial | ZNF521 |
| 2.77675979 | 0.857 | 0.102 | 0 | Lymphatic Endothelial | LAMA4 |
| 2.77077765 | 0.878 | 0.155 | 0 | Lymphatic Endothelial | NR2F2-AS1 |
| 2.74305139 | 0.579 | 0.023 | 0 | Lymphatic Endothelial | SYT1 |
| 2.73714741 | 0.681 | 0.029 | 0 | Lymphatic Endothelial | NALCN |
| 2.71539914 | 0.722 | 0.003 | 0 | Lymphatic Endothelial | FLT4 |
| 2.69439057 | 0.857 | 0.091 | 0 | Lymphatic Endothelial | COLEC12 |
| 2.6790771 | 0.597 | 0.019 | 0 | Lymphatic Endothelial | TLL1 |
| 2.63839586 | 0.657 | 0.016 | 0 | Lymphatic Endothelial | NR2F1-AS1 |
| 4.90560416 | 0.763 | 0.064 | 0 | Mast Cells | NTM |
| 4.76039079 | 0.9 | 0.002 | 0 | Mast Cells | CPA3 |
| 4.43444342 | 0.819 | 0.012 | 0 | Mast Cells | HPGD |
| 4.40141192 | 0.884 | 0.002 | 0 | Mast Cells | KIT |
| 4.06469981 | 0.855 | 0.069 | 0 | Mast Cells | STX3 |
| 3.96471554 | 0.819 | 0.001 | 0 | Mast Cells | HDC |
| 3.94671378 | 0.795 | 0.002 | 0 | Mast Cells | MS4A2 |
| 3.84859005 | 0.743 | 0.01 | 0 | Mast Cells | IL18R1 |
| 3.76377057 | 0.731 | 0.004 | 0 | Mast Cells | RAB27B |
| 3.75729715 | 0.783 | 0.023 | 0 | Mast Cells | HPGDS |
| 3.64674922 | 0.747 | 0.073 | 0 | Mast Cells | MEIS2 |
| 3.59711351 | 0.88 | 0.225 | 3.15E-205 | Mast Cells | BMP2K |
| 3.51941131 | 0.691 | 0.008 | 0 | Mast Cells | CDK15 |
| 3.49745174 | 0.751 | 0.023 | 0 | Mast Cells | ALOX5 |
| 3.46577517 | 0.502 | 0.01 | 0 | Mast Cells | LINC02147 |
| 3.40495436 | 0.767 | 0.069 | 0 | Mast Cells | BACE2 |
| 3.37527028 | 0.964 | 0.371 | 1.59E-175 | Mast Cells | SLC24A3 |
| 3.32225361 | 0.703 | 0.01 | 0 | Mast Cells | MCTP2 |
| 3.27922936 | 0.843 | 0.07 | 0 | Mast Cells | ARHGAP15 |
| 3.27120623 | 0.703 | 0.062 | 0 | Mast Cells | SGK1 |
| 3.25872722 | 0.635 | 0.013 | 0 | Mast Cells | KIAA1549 |
| 3.24281482 | 0.888 | 0.187 | 1.83E-223 | Mast Cells | ELMO1 |
| 3.20650938 | 0.695 | 0.051 | 0 | Mast Cells | ST8SIA1 |
| 3.18993484 | 0.627 | 0.001 | 0 | Mast Cells | TPSB2 |
| 3.17818409 | 0.643 | 0.023 | 0 | Mast Cells | SKAP1 |
| 3.13274014 | 0.606 | 0.004 | 0 | Mast Cells | GATA2 |
| 3.1164897 | 0.594 | 0.005 | 0 | Mast Cells | RGS13 |
| 3.09355811 | 0.578 | 0.011 | 0 | Mast Cells | SLC18A2 |
| 3.08856867 | 0.643 | 0.01 | 0 | Mast Cells | VWA5A |
| 3.07598124 | 0.783 | 0.252 | 1.48E-136 | Mast Cells | SYTL3 |
| 3.05434935 | 0.699 | 0.169 | 1.19E-136 | Mast Cells | LRP1B |
| 3.01661037 | 0.727 | 0.089 | 4.67E-290 | Mast Cells | CHST11 |
| 3.00787946 | 0.811 | 0.179 | 6.03E-190 | Mast Cells | ANKRD44 |
| 2.96714212 | 0.835 | 0.327 | 6.97E-121 | Mast Cells | FER |
| 2.91574607 | 0.602 | 0.053 | 0 | Mast Cells | SLCO2B1 |
| 2.89459565 | 0.586 | 0.016 | 0 | Mast Cells | TOX |
| 2.85602336 | 0.59 | 0.048 | 0 | Mast Cells | CPM |
| 2.83349071 | 0.578 | 0.002 | 0 | Mast Cells | RHEX |
| 2.8236518 | 0.691 | 0.118 | 2.55E-194 | Mast Cells | CD44 |
| 2.8138847 | 0.582 | 0.04 | 0 | Mast Cells | PPM1H |
| 2.79927469 | 0.59 | 0.03 | 0 | Mast Cells | KCNQ1 |
| 2.79675862 | 0.663 | 0.089 | 2.07E-243 | Mast Cells | ACER3 |
| 2.79330925 | 0.92 | 0.199 | 1.17E-208 | Mast Cells | LRMDA |
| 2.78568836 | 0.57 | 0.033 | 0 | Mast Cells | PRKCB |
| 2.77057572 | 0.418 | 0.002 | 0 | Mast Cells | AC092979.1 |
| 2.74207108 | 0.53 | 0.092 | 2.37E-140 | Mast Cells | LMO4 |
| 2.72313604 | 0.462 | 0.009 | 0 | Mast Cells | ENPP3 |
| 2.68334349 | 0.59 | 0.08 | 4.13E-203 | Mast Cells | STXBP6 |
| 2.68030745 | 0.582 | 0.047 | 0 | Mast Cells | SYTL2 |
| 2.67541307 | 0.49 | 0.002 | 0 | Mast Cells | P2RX1 |
| 5.36782924 | 0.976 | 0.106 | 0 | Adipocytes | GPAM |
| 5.11736617 | 0.964 | 0.037 | 0 | Adipocytes | PDE3B |
| 4.88301091 | 0.976 | 0.047 | 0 | Adipocytes | PPARG |
| 3.92215437 | 0.982 | 0.009 | 0 | Adipocytes | PLIN1 |
| 3.91954907 | 0.911 | 0.109 | 3.42E-271 | Adipocytes | GRK3 |
| 3.80091171 | 0.976 | 0.104 | 0 | Adipocytes | LAMA4 |
| 3.69602352 | 0.94 | 0.046 | 0 | Adipocytes | MGST1 |
| 3.5616431 | 0.464 | 0.004 | 0 | Adipocytes | SCD |
| 3.54280225 | 0.94 | 0.126 | 2.87E-268 | Adipocytes | PLIN4 |
| 3.49705007 | 0.982 | 0.19 | 2.06E-200 | Adipocytes | SOX5 |
| 3.4906178 | 0.923 | 0.032 | 0 | Adipocytes | TMEM132C |
| 3.47560177 | 0.869 | 0.125 | 1.48E-211 | Adipocytes | LPL |
| 3.407356 | 0.988 | 0.201 | 3.20E-188 | Adipocytes | EBF1 |
| 3.36884315 | 0.839 | 0.058 | 0 | Adipocytes | LIPE-AS1 |
| 3.32632377 | 0.994 | 0.558 | 4.05E-108 | Adipocytes | ACACB |
| 3.1786584 | 0.893 | 0.052 | 0 | Adipocytes | APBB1IP |
| 3.15717126 | 0.631 | 0.009 | 0 | Adipocytes | FASN |
| 3.11706215 | 0.833 | 0.039 | 0 | Adipocytes | PLXNA4 |
| 3.11107694 | 0.857 | 0.002 | 0 | Adipocytes | ADIPOQ |
| 3.10847807 | 0.839 | 0.009 | 0 | Adipocytes | LINC02237 |
| 3.04258436 | 0.905 | 0.355 | 1.10E-86 | Adipocytes | ACSL1 |
| 2.9869413 | 0.768 | 0.042 | 0 | Adipocytes | LINC00598 |
| 2.95486347 | 0.774 | 0.007 | 0 | Adipocytes | PTGER3 |
| 2.88887027 | 0.881 | 0.048 | 0 | Adipocytes | CPM |
| 2.86773012 | 0.881 | 0.067 | 0 | Adipocytes | ADH1B |
| 2.84045137 | 0.952 | 0.318 | 5.78E-105 | Adipocytes | EHBP1 |
| 2.83304415 | 0.792 | 0.014 | 0 | Adipocytes | PRKAR2B |
| 2.82068605 | 0.905 | 0.134 | 3.54E-212 | Adipocytes | SLC16A7 |
| 2.80565482 | 0.929 | 0.18 | 6.67E-174 | Adipocytes | MLXIPL |
| 2.74118191 | 0.893 | 0.103 | 1.06E-275 | Adipocytes | PNPLA2 |
| 2.69133986 | 0.702 | 0.024 | 0 | Adipocytes | AC002066.1 |
| 2.69079183 | 0.774 | 0.051 | 0 | Adipocytes | MAOA |
| 2.67890171 | 0.887 | 0.04 | 0 | Adipocytes | PECR |
| 2.67344819 | 0.935 | 0.164 | 8.32E-187 | Adipocytes | DIRC3 |
| 2.66557903 | 0.708 | 0.01 | 0 | Adipocytes | FABP4 |
| 2.64797424 | 0.929 | 0.158 | 1.40E-205 | Adipocytes | AQP7 |
| 2.62279373 | 0.952 | 0.394 | 2.96E-99 | Adipocytes | SIK2 |
| 2.61782189 | 0.679 | 0.046 | 0 | Adipocytes | ELOVL5 |
| 2.6026313 | 0.887 | 0.173 | 5.74E-161 | Adipocytes | MAST4 |
| 2.59754817 | 0.958 | 0.476 | 1.06E-97 | Adipocytes | TNS1 |
| 2.5681249 | 0.851 | 0.022 | 0 | Adipocytes | CARMN |
| 2.56075735 | 0.863 | 0.106 | 5.28E-215 | Adipocytes | GPC6 |
| 2.54925524 | 0.899 | 0.231 | 2.04E-122 | Adipocytes | VKORC1L1 |
| 2.53267729 | 0.786 | 0.002 | 0 | Adipocytes | GYG2 |
| 2.52606078 | 0.685 | 0.015 | 0 | Adipocytes | ADRA1A |
| 2.51685927 | 0.69 | 0.104 | 2.88E-142 | Adipocytes | CLSTN2 |
| 2.51179905 | 0.792 | 0.052 | 0 | Adipocytes | TRHDE |
| 2.50294951 | 0.732 | 0.023 | 0 | Adipocytes | LIPE |
| 2.45768721 | 0.554 | 0.035 | 7.56E-279 | Adipocytes | AC104574.2 |
| 2.45121823 | 0.893 | 0.368 | 1.11E-74 | Adipocytes | EEPD1 |
